# Supplementary material for: Multimodal Machine Learning-based Knee Osteoarthritis Progression Prediction from Plain Radiographs and Clinical Data
Source: Sci Rep. 2019 Dec 27;9:20038. doi: 10.1038/s41598-019-56527-3 (PMC6934728; doi:10.1038/s41598-019-56527-3)
Supplement: Supplementary file 1 — Supplementary Information [file 41598_2019_56527_MOESM1_ESM.pdf]

Multimodal Machine Learning-based Knee Osteoarthritis Progression Prediction from Plain Radiographs and Clinical Data: Supplementary Information

Aleksei Tiulpin[1,8,*], Stefan Klein[2], Sita M.A. Bierma-Zeinstra[3,4], Jérôme Thevenot[1], Esa Rahtu[5], Joyce van Meurs[6], Edwin H.G. Oei[7], and Simo Saarakkala[1,8]

[1]Research Unit of Medical Imaging, Physics and Technology, University of Oulu, Oulu, Finland.
[2]Biomedical Imaging Group Rotterdam, Depts. of Medical Informatics & Radiology, Erasmus MC, University Medical Center Rotterdam, the Netherlands.
[3]Department of General Practice, Erasmus MC, University Medical Center Rotterdam, the Netherlands
[4]Department of Orthopedics, Erasmus MC, University Medical Center Rotterdam, the Netherlands.
[5]Department of Signal Processing, University of Tampere, Tampere, Finland.
[6]Department of Internal Medicine, Erasmus MC, University Medical Center Rotterdam, the Netherlands
[7]Department of Radiology & Nuclear Medicine, University Medical Center Rotterdam, the Netherlands
[8]Department of Diagnostic Radiology, Oulu University Hospital, Oulu, Finland
[*]aleksei.tiulpin@oulu.fi

1 Supplementary Experiments

Fixed Joint Space Width versus Our Approach

To validate whether our method provides any additional value, we also compared it to imaging biomarkers more sensitive than KL-grades. As such, we validated our approach against fixed Joint Space Width (fJSW) measurements[1] available in OAI dataset and validated the following models:

1. GBM model that uses Age, Sex, Body-Mass Index (BMI), total Western Ontario and McMaster Universities Arthritis Index (WOMAC) score, injury and surgery history (model S1).

2. Model S1 with the addition of a KL grade (model 4 in the main text).

3. Model S1 with the addition of fJSW measurements (model S2).

4. Model S2 with the addition of fJSW measurements (model S3).

5. Model 6 in the main text.

6. Model 7 in the main text.

The experiments were conducted as follows. As mentioned previously, we leveraged the existing fJSW measurements for our data from the train set (OAI). MOST dataset was not used as the fJSW measurements are not available for it. To simulate the independent testing, we kept one data acquisition site out in an external cross-validation loop and trained our model exactly as described in Methods using the remaining data and 5-fold cross-validation. After the training was finished, we performed prediction on the data which was kept out in the external cross-validation loop and computed the performance metric. This procedure was conducted for every data acquisition site in OAI dataset (5 sites in total) and we eventually averaged the results across the data acquisition sites. The results of the experiment are presented in Supplementary Table S1.

Supplementary Table S1 shows that the model which performed the best (model 7) in the main experiments also outperformed all the models which included fJSW measurements – models S1, S2 and S3, respectively. A secondary observation from the conducted experiment on OAI dataset is that the performance of all the methods differed from the MOST dataset. We point out that these datasets are different (e.g. in percentage of progressors, see Table S3), therefore the performance between them cannot be compared directly. Despite this, all the conclusions in our study still hold as shown in Supplementary Table S1.

1.1 Optimal Train Dataset Size

In this experiment, we investigated the relationship between the performance of our Convolutional Neural Network (CNN) on the test set and the size of the training data. Specifically, we sampled 400, 800, 1600 and 3200 knee images from the train set so that the each sample has exactly the same distribution of progressors and non-progressors. Subsequently, we trained our CNN exactly as described in Methods and evaluated average precision on the test set. These results are shown in Supplementary Figure S3. From this figure, it can be observed that the performance of our our model on the test set increases with the increase of training data.

1.2 Feature Importance of the Second-Level Model

We utilized two techniques for getting the insights about the contributions of each of the factors used in models 6 and 7 in the main text to the decision. Specifically, we used Shapley Additive Explanation (SHAP) technique[2] to explore the feature importance on the test set. We also used the relative predictor importance information naturally available from GBM after training[3].

The train (Supplementary Figure S4) and the test (Supplementary Figure S5) feature importance plots indicate that the predictions produced by our model have highest contributions into the decisions produced by both models 6 and 7, respectively. Interestingly, both train and test feature importance plots indicate the importance of the symptomatic assessment for the final prediction (Western Ontario and McMaster Universities Arthritis Index, WOMAC[4])

References

1. Neumann, G. *et al.* Location specific radiographic joint space width for osteoarthritis progression. *Osteoarthr. cartilage* **17**, 761–765 (2009).

2. Lundberg, S. M., Erion, G. G. & Lee, S.-I. Consistent individualized feature attribution for tree ensembles. *arXiv preprint arXiv:1802.03888* (2018).

3. Hastie, T., Tibshirani, R., Friedman, J. & Franklin, J. The elements of statistical learning: data mining, inference and prediction. *The Math. Intell.* **27**, 83–85 (2005).

4. Bellamy, N., Buchanan, W. W., Goldsmith, C. H., Campbell, J. & Stitt, L. W. Validation study of womac: a health status instrument for measuring clinically important patient relevant outcomes to antirheumatic drug therapy in patients with osteoarthritis of the hip or knee. *The J. rheumatology* **15**, 1833–1840 (1988).

2 Supplementary Data

Table S1. Assessments of added value of our method compared to semi-automatic measurements of fixed Joint Space Width (fJSW). We used the data from OAI dataset and conducted experiments with nested cross-validation, keeping one data acquisition site out from the dataset and re-training out method and the models described below on the remaining parts. The results in the table show the average performances across data acquisition sites in OAI dataset and the standard deviation.

Model #	Model	AUC	AP
S1	Age, Sex, BMI, Injury, Surgery, WOMAC (GBM)	0.63±0.04	0.55±0.04
4	Age, Sex, BMI, Injury, KL-grade, Surgery, WOMAC (GBM)	0.69±0.03	0.61±0.06
S2	Age, Sex, BMI, Injury, Surgery, WOMAC, fJSW at all locations (GBM)	0.67 ±0.03	0.61±0.05
S3	Age, Sex, BMI, Injury, KL-grade, Surgery, WOMAC, fJSW at all locations (GBM)	0.73±0.03	0.66±0.07
6	CNN + Age, Sex, BMI, Injury, Surgery, WOMAC (GBM-based fusion)	0.70±0.04	0.64±0.05
7	CNN + Age, Sex, BMI, Injury, Surgery, WOMAC, KL-grade (GBM-based fusion)	<u>0.75±0.03</u>	<u>0.69±0.06</u>

KL-grade – Kellgren-Lawrence grade
CNN – Deep Convolutional Neural Network
BMI – Body-Mass Index
WOMAC – Western Ontario and McMaster Universities Arthritis Index
AUC – Area Under the Receiver Operating Characteristic Curve
AP – Average Precision
GBM – Gradient Boosting Machine

Table S2. Subject-level characteristics for subsets of Osteoarthritis Initiative (OAI) and Multicenter Osteoarthritis Study (MOST) datasets, used in our work as train and test sets, respectively.

Dataset	Age	BMI	# Females	# Males
OAI (Train)	61.16±9.19	28.62±4.84	1,552	1,159
MOST (Test)	62.50±8.11	30.74±5.97	1,303	826

BMI – Body Mass Index

Table S3. Knee-level characteristics for subsets of Osteoarthritis Initiative (OAI) and Multicenter Osteoarthritis Study (MOST) datasets used in this study as train and test sets, respectively. KL-0 to KL4 represent Kellgren-Lawrence Grading scale of osteoarthritis (OA) – from healthy knee to end-stage OA. Here, (P) indicates the knees which progressed during the follow-up visits and (NP) the ones which did not progress.

**Dataset**	**Subset**	**KL-grade**					**Total**	**# Left**	**# Right**
		0	1	2	3	4			
OAI	NP	2,133	702	569	193	0	3,597	1,803	1,794
	P	271	466	346	248	0	1,331	654	677
MOST	NP	1,558	336	314	209	0	2,417	1,208	1,209
	P	322	387	380	412	0	1,501	716	785

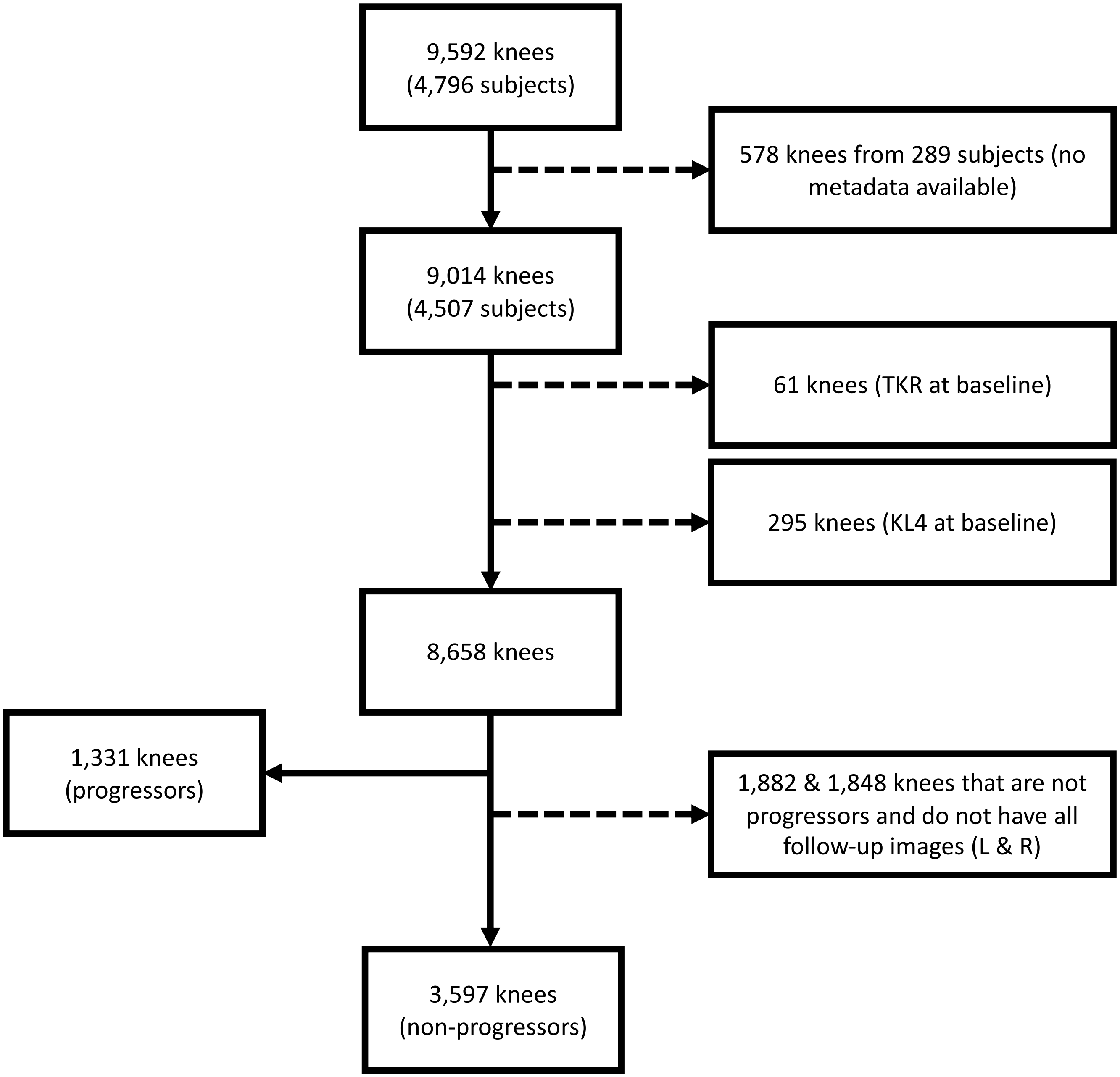

Figure S1. Data selection flowchart for Osteoarthritis Initiative (OAI) dataset which was used to train the model.

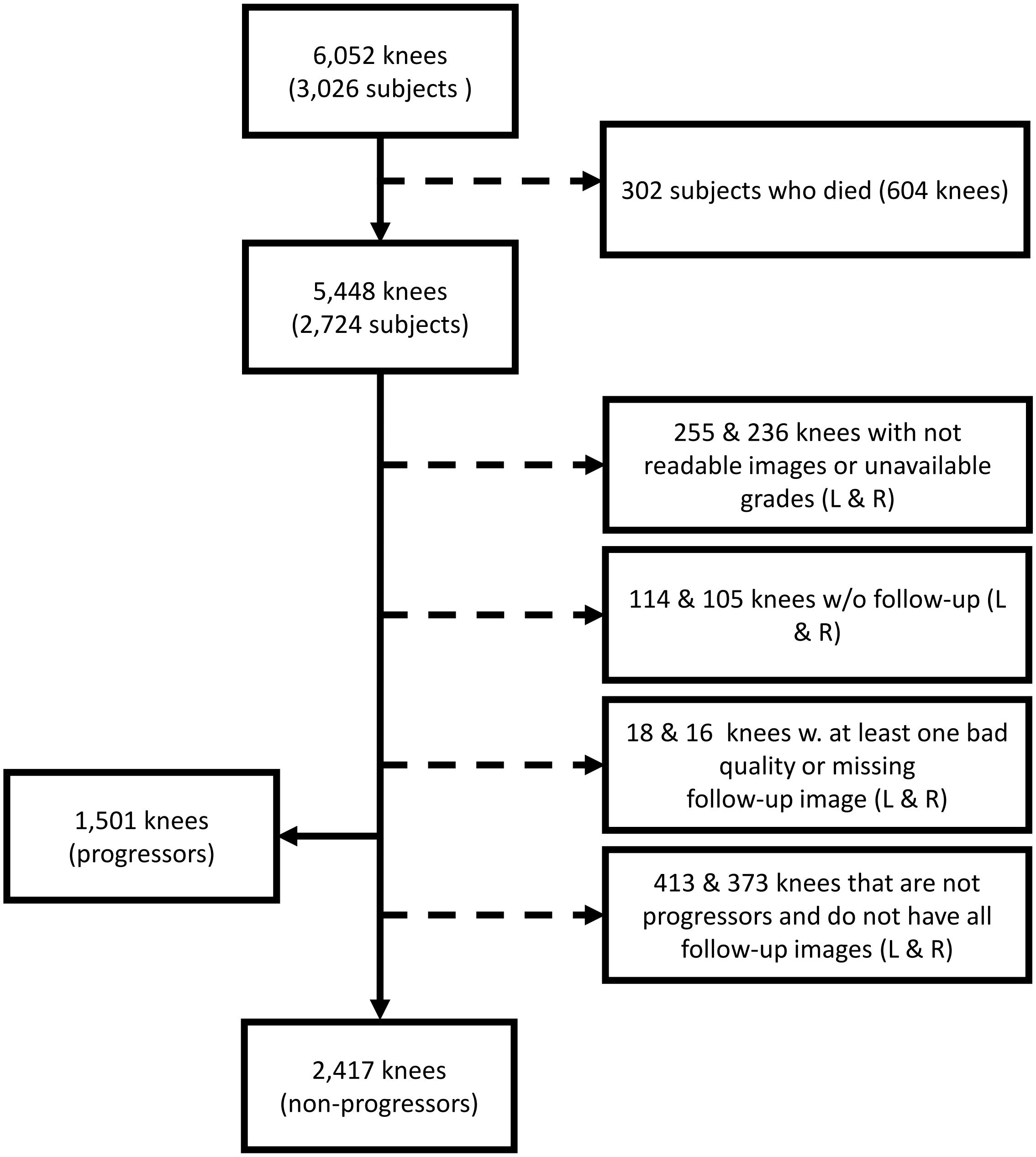

Figure S2. Data selection flowchart for Multicenter Osteoarthritis Study (MOST) dataset which was used to test the model.

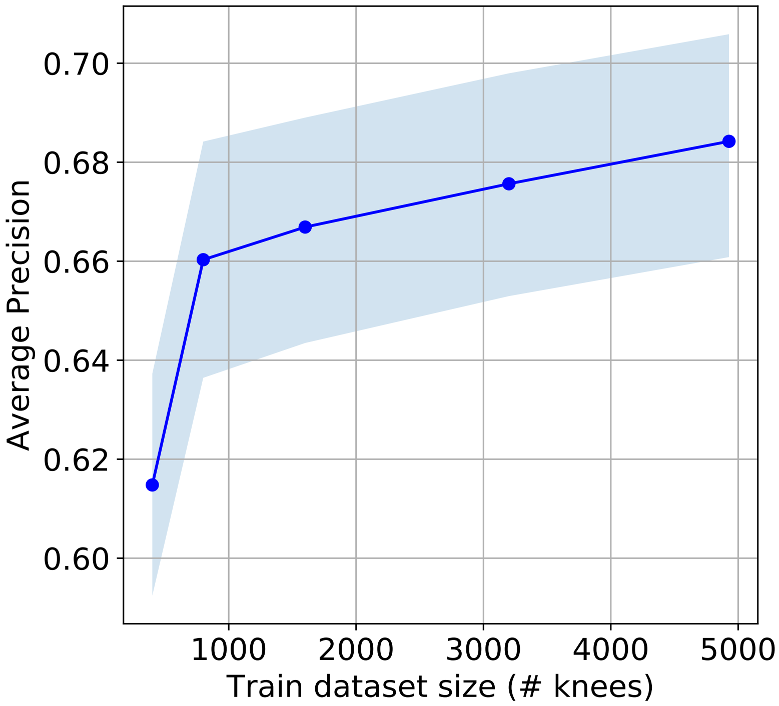

Figure S3. Learning curve showing the performance of the convolutional neural network on the test set derived from Multicenter Osteoarthritis Study dataset. 95% confidence intervals computed via stratified bootstrapping are also highlighted. The smallest dataset in this experiment corresponded to 400 knees and the largest dataset corresponded to the experiments shown in the main text. The prevalence of progressors in each of the sub-sampled datasets corresponds to the prevalence in the whole train set.

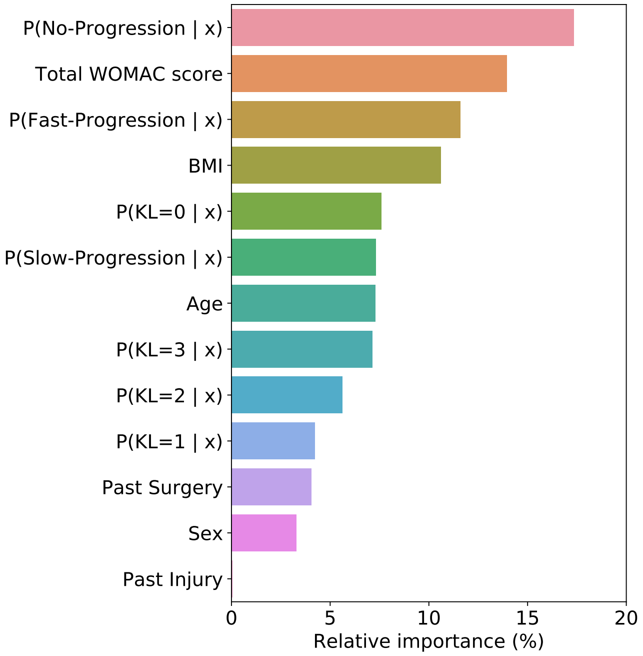

(a) Model without KL-grade

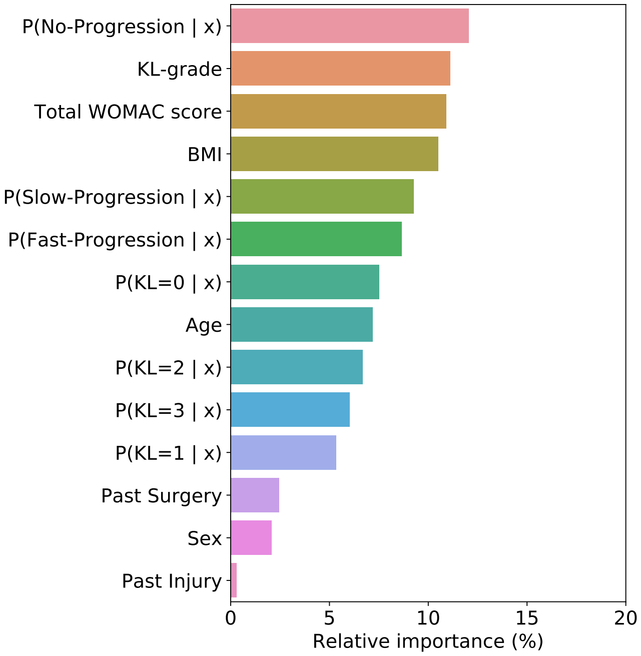

(b) Model with KL-grade

Figure S4. Average relative feature importance derived from training GBM (LightGBM implementation) on Osteoarthritis Initiative dataset. We computed feature importance per each training fold and averaged them across the folds.

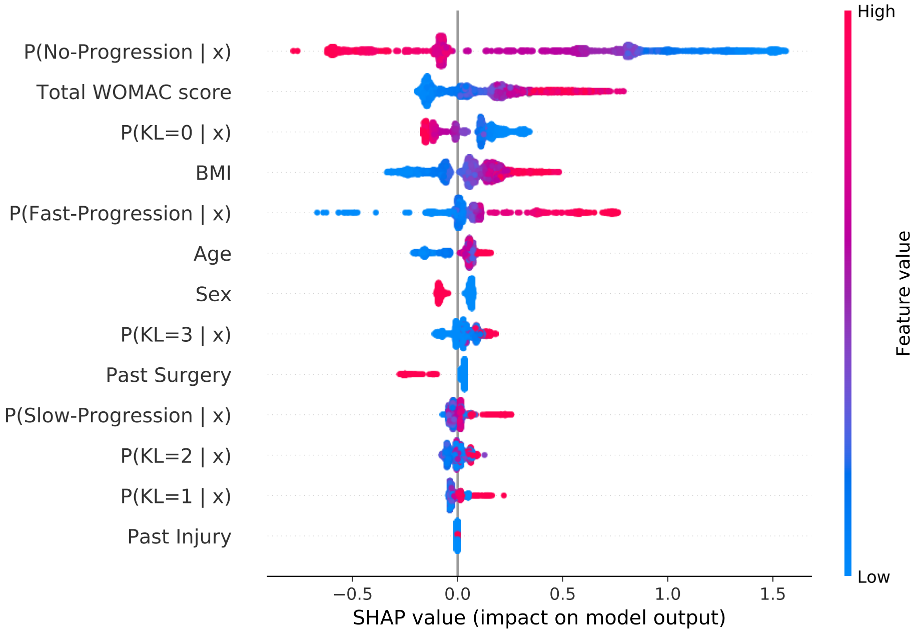

(a) Model 6 (ensemble w/o KL-grade)

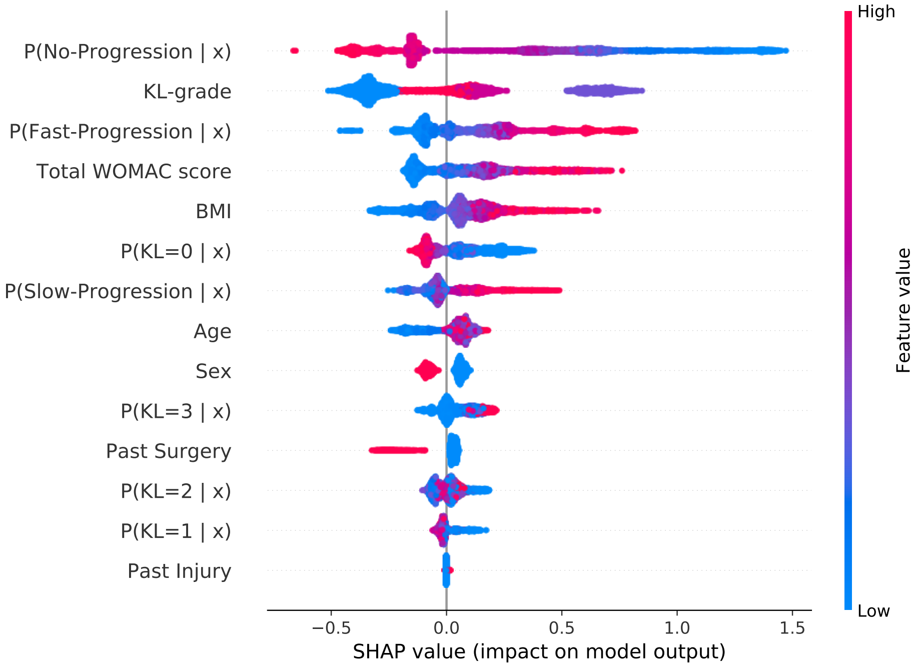

(b) Model 7 (ensemble with KL-grade)

Figure S5. SHAP feature importance on the test set derived from Multicenter Osteoarthritis Study. Here, the model output indicates $P(progressor|x)$, where x is a model input.

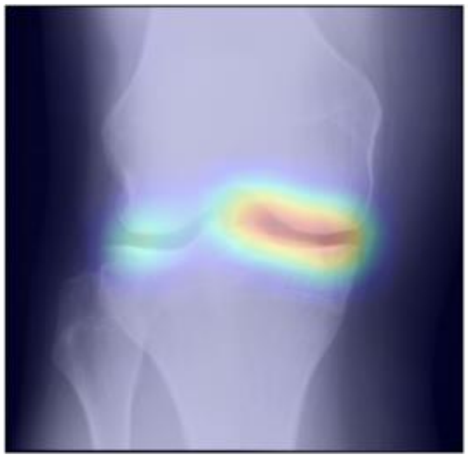

(a) KL-0 to KL-2, slow

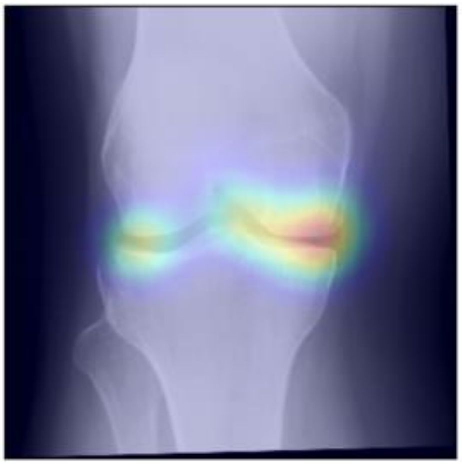

(b) KL-0 to KL-3, slow

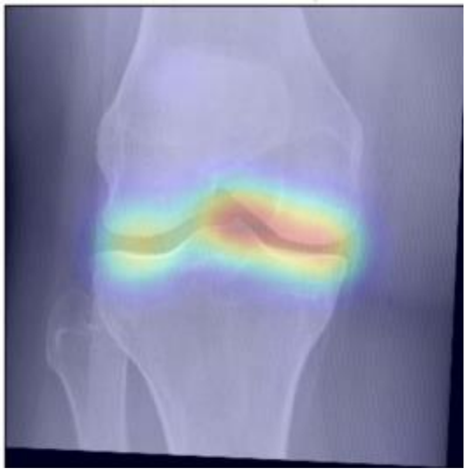

(c) KL-0 to KL2, slow

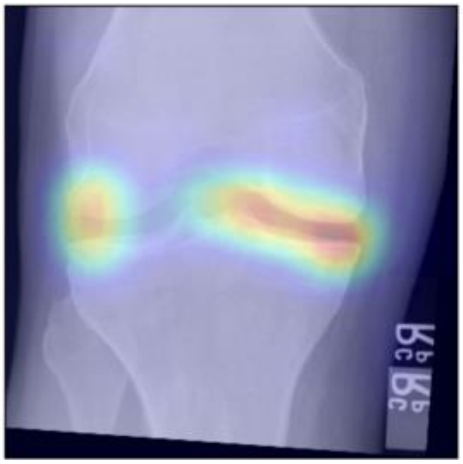

(d) KL-1 to KL-3, slow

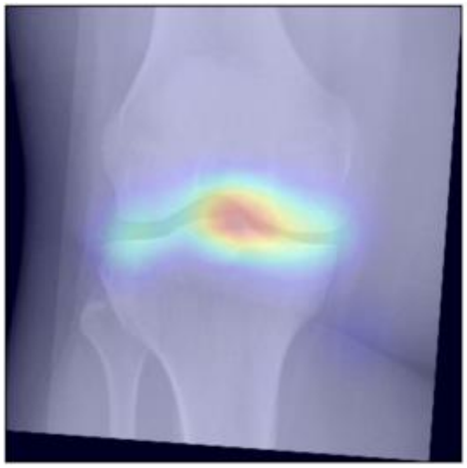

(e) KL-1 to KL-2, fast

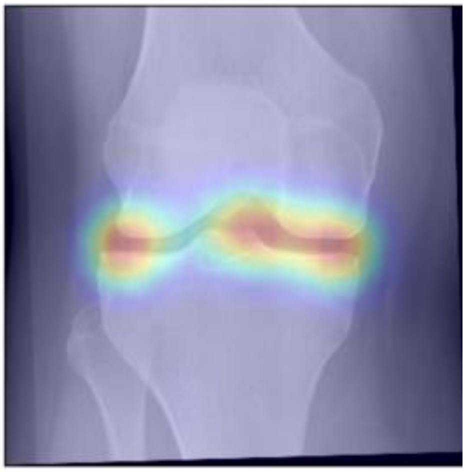

(f) KL-1 to KL3, fast

Figure S6. Examples of GradCAM-based attention maps for the knees progressed from no osteoarthritis to osteoarthritis. Fine-grained sub-types of progression are also specified. The presented images are of 140×140 mm.

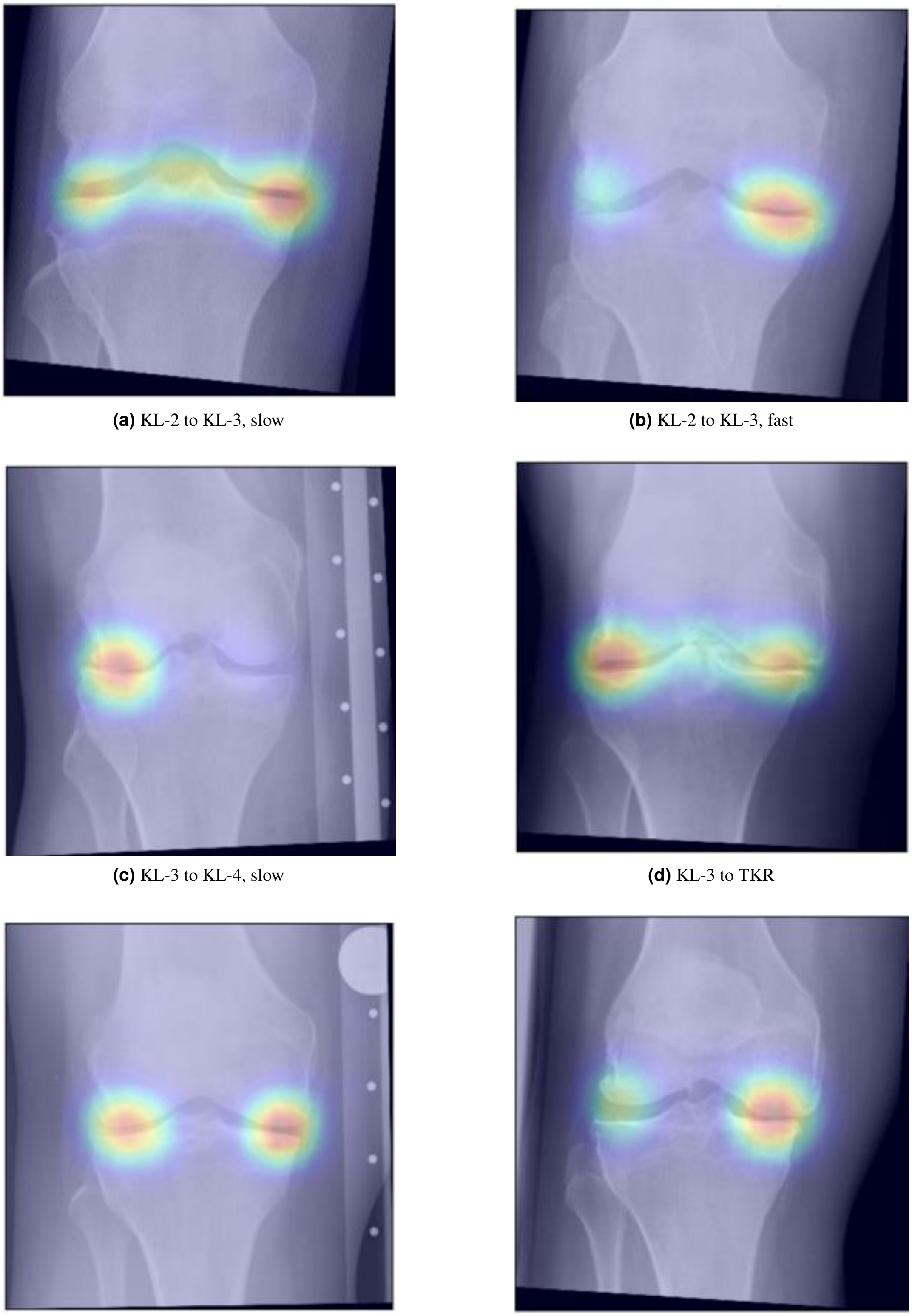

(a) KL-2 to KL-3, slow

(b) KL-2 to KL-3, fast

(c) KL-3 to KL-4, slow

(d) KL-3 to TKR

(e) KL-2 to KL-3, fast

(f) KL-3 to KL-4, fast

Figure S7. Examples of GradCAM-based attention maps for the knees having osteoarthritis at baseline and progressed in the future. Fine-grained sub-types of progression are also specified. The presented images are of 140×140 mm.

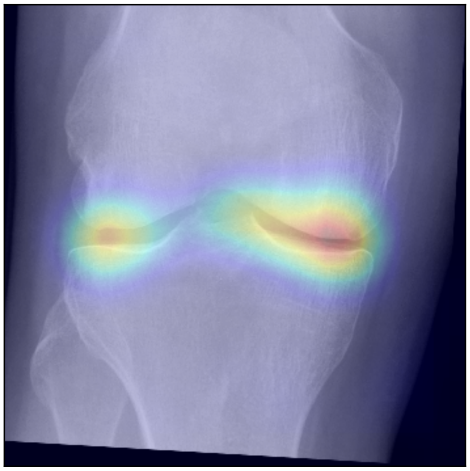

(a) KL-1

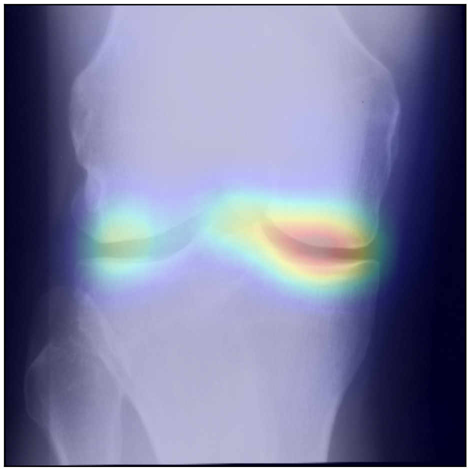

(b) KL-0

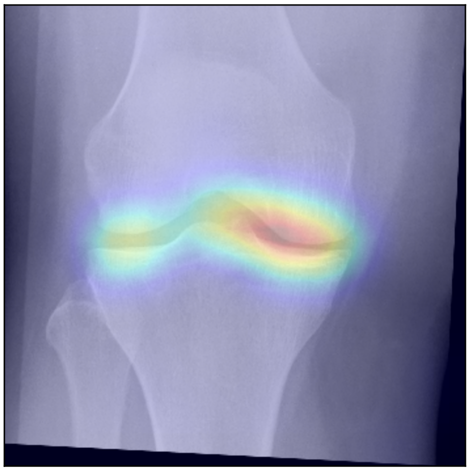

(c) KL-1

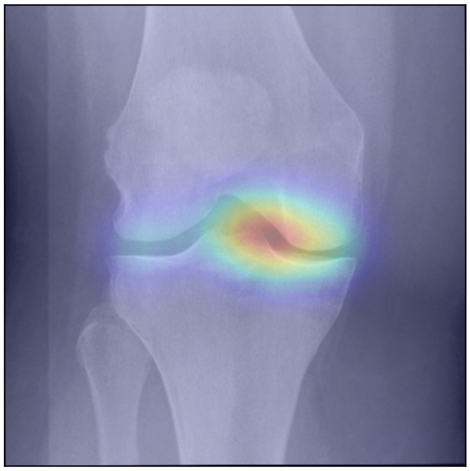

(d) KL-0

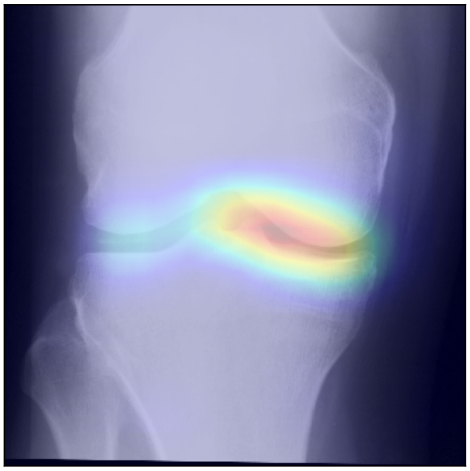

(e) KL-1

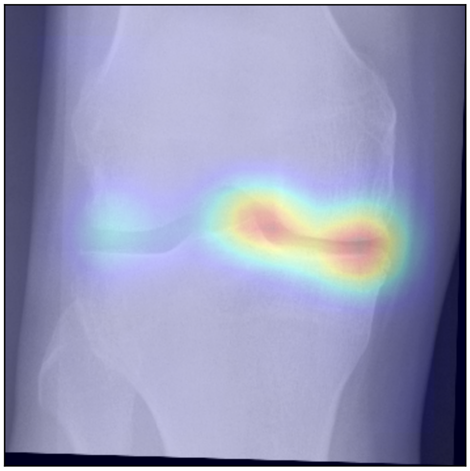

(f) KL-1

Figure S8. Examples of GradCAM-based attention maps for the knees having no osteoarthritis at baseline and that did progress within the next 7 years. Baseline Kellgren-Lawrence (KL) grades are specified. The presented images are of 140×140 mm.

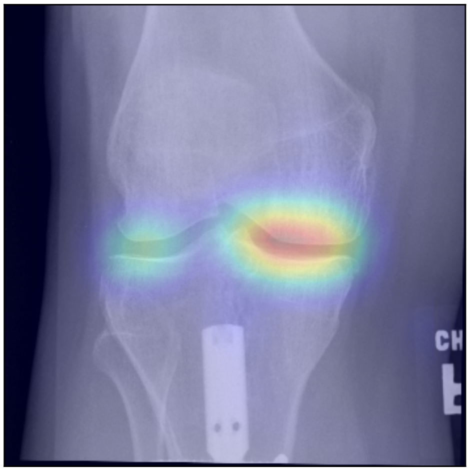

(a) KL-2

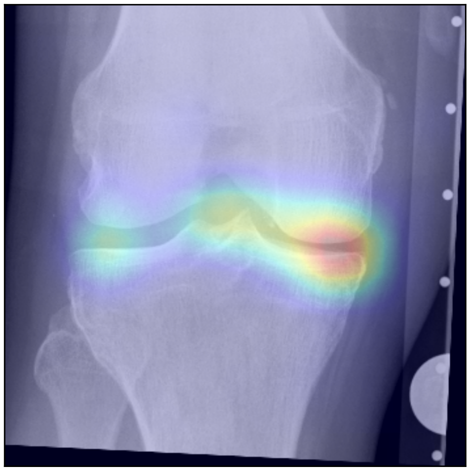

(b) KL-2

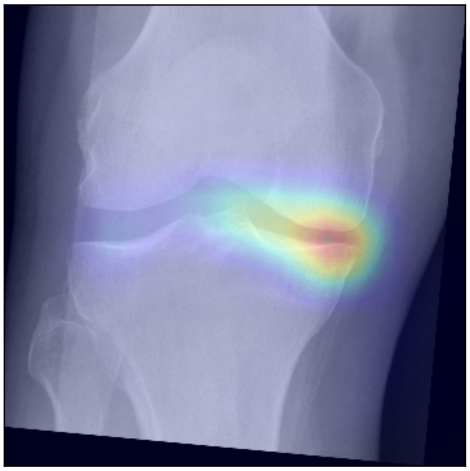

(c) KL-2

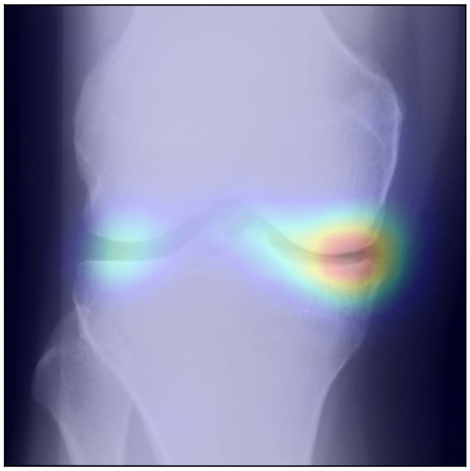

(d) KL-2

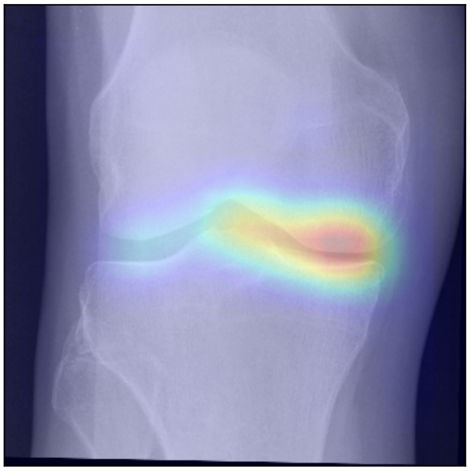

(e) KL-2

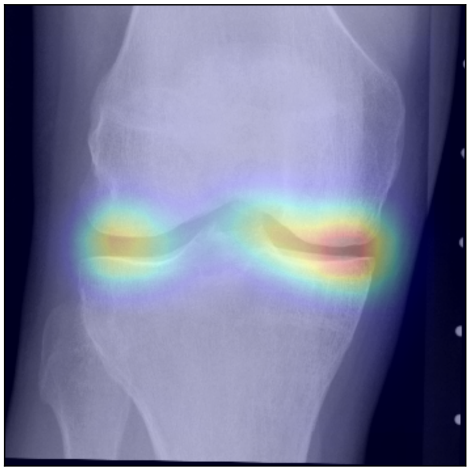

(f) KL-2

Figure S9. Examples of GradCAM-based attention maps for the knees having early osteoarthritis at the baseline and that did not progress withing the next 7 years. Baseline Kellgren-Lawrence (KL) grades are specified. The presented images are of 140×140 mm.
